# The Phospholipid Flippase ATP8B1 is Involved in the Pathogenesis of Ulcerative Colitis via Establishment of Intestinal Barrier Function

**DOI:** 10.1093/ecco-jcc/jjae024

**Published:** 2024-02-16

**Authors:** Pim J Koelink, Valentina E Gómez-Mellado, Suzanne Duijst, Manon van Roest, Sander Meisner, Kam S Ho-Mok, Sabrina Frank, Babette S Appelman, Lysbeth ten Bloemendaal, Georg F Vogel, Stan F J van de Graaf, Piter J Bosma, Ronald P J Oude Elferink, Manon E Wildenberg, Coen C Paulusma

**Affiliations:** Amsterdam University Medical Centers, University of Amsterdam, Tytgat Institute for Liver and Intestinal Research, Amsterdam, The Netherlands; Amsterdam Gastroenterology Endocrinology Metabolism, Amsterdam, The Netherlands; Amsterdam University Medical Centers, University of Amsterdam, Tytgat Institute for Liver and Intestinal Research, Amsterdam, The Netherlands; Amsterdam Gastroenterology Endocrinology Metabolism, Amsterdam, The Netherlands; Amsterdam University Medical Centers, University of Amsterdam, Tytgat Institute for Liver and Intestinal Research, Amsterdam, The Netherlands; Amsterdam Gastroenterology Endocrinology Metabolism, Amsterdam, The Netherlands; Amsterdam University Medical Centers, University of Amsterdam, Tytgat Institute for Liver and Intestinal Research, Amsterdam, The Netherlands; Amsterdam Gastroenterology Endocrinology Metabolism, Amsterdam, The Netherlands; Amsterdam University Medical Centers, University of Amsterdam, Tytgat Institute for Liver and Intestinal Research, Amsterdam, The Netherlands; Amsterdam Gastroenterology Endocrinology Metabolism, Amsterdam, The Netherlands; Amsterdam University Medical Centers, University of Amsterdam, Tytgat Institute for Liver and Intestinal Research, Amsterdam, The Netherlands; Amsterdam Gastroenterology Endocrinology Metabolism, Amsterdam, The Netherlands; Amsterdam University Medical Centers, University of Amsterdam, Tytgat Institute for Liver and Intestinal Research, Amsterdam, The Netherlands; Amsterdam University Medical Centers, University of Amsterdam, Tytgat Institute for Liver and Intestinal Research, Amsterdam, The Netherlands; Amsterdam University Medical Centers, University of Amsterdam, Tytgat Institute for Liver and Intestinal Research, Amsterdam, The Netherlands; Amsterdam Gastroenterology Endocrinology Metabolism, Amsterdam, The Netherlands; Department of Paediatrics I, Medical University of Innsbruck, 6020 Innsbruck, Austria; Institute of Cell Biology, Biocenter, Medical University of Innsbruck, 6020 Innsbruck, Austria; Amsterdam University Medical Centers, University of Amsterdam, Tytgat Institute for Liver and Intestinal Research, Amsterdam, The Netherlands; Amsterdam Gastroenterology Endocrinology Metabolism, Amsterdam, The Netherlands; Department of Gastroenterology and Hepatology, Amsterdam University Medical Centers, University of Amsterdam, Amsterdam, The Netherlands; Amsterdam University Medical Centers, University of Amsterdam, Tytgat Institute for Liver and Intestinal Research, Amsterdam, The Netherlands; Amsterdam Gastroenterology Endocrinology Metabolism, Amsterdam, The Netherlands; Department of Gastroenterology and Hepatology, Amsterdam University Medical Centers, University of Amsterdam, Amsterdam, The Netherlands; Amsterdam University Medical Centers, University of Amsterdam, Tytgat Institute for Liver and Intestinal Research, Amsterdam, The Netherlands; Amsterdam Gastroenterology Endocrinology Metabolism, Amsterdam, The Netherlands; Department of Gastroenterology and Hepatology, Amsterdam University Medical Centers, University of Amsterdam, Amsterdam, The Netherlands; Amsterdam University Medical Centers, University of Amsterdam, Tytgat Institute for Liver and Intestinal Research, Amsterdam, The Netherlands; Amsterdam Gastroenterology Endocrinology Metabolism, Amsterdam, The Netherlands; Department of Gastroenterology and Hepatology, Amsterdam University Medical Centers, University of Amsterdam, Amsterdam, The Netherlands; Amsterdam University Medical Centers, University of Amsterdam, Tytgat Institute for Liver and Intestinal Research, Amsterdam, The Netherlands; Amsterdam Gastroenterology Endocrinology Metabolism, Amsterdam, The Netherlands

**Keywords:** IBD, Ulcerative Colitis, Intestinal Epithelial Barrier, ATP8B1, CLDN4

## Abstract

**Aims:**

Patients with mutations in *ATP8B1* develop progressive familial intrahepatic cholestasis type 1 [PFIC1], a severe liver disease that requires life-saving liver transplantation. PFIC1 patients also present with gastrointestinal problems, including intestinal inflammation and diarrhoea, which are aggravated after liver transplantation. Here we investigate the intestinal function of ATP8B1 in relation to inflammatory bowel diseases.

**Methods:**

*ATP8B1* expression was investigated in intestinal samples of patients with Crohn’s disease [CD] or ulcerative colitis [UC] as well as in murine models of intestinal inflammation. Colitis was induced in ATP8B1-deficient mice with dextran sodium sulphate [DSS] and intestinal permeability was investigated. Epithelial barrier function was assessed in ATP8B1 knockdown Caco2-BBE cells. Co-immunoprecipitation experiments were performed in Caco2-BBE cells overexpressing ATP8B1-eGFP. Expression and localization of ATP8B1 and tight junction proteins were investigated in cells and in biopsies of UC and PFIC1 patients.

**Results:**

ATP8B1 expression was decreased in UC and DSS-treated mice, and was associated with a decreased tight junctional pathway transcriptional programme. ATP8B1-deficient mice were extremely sensitive to DSS-induced colitis, as evidenced by increased intestinal barrier leakage. ATP8B1 knockdown cells showed delayed barrier establishment that affected Claudin-4 [CLDN4] levels and localization. CLDN4 immunohistochemistry showed a tight junctional staining in control tissue, whereas in UC and intestinal PFIC1 samples, CLDN4 was not properly localized.

**Conclusion:**

ATP8B1 is important in the establishment of the intestinal barrier. Downregulation of ATP8B1 levels in UC, and subsequent altered localization of tight junctional proteins, including CLDN4, might therefore be an important mechanism in UC pathophysiology.

## 1. Introduction

ATP8B1 is a phospholipid flippase belonging to the P4-ATPase family of proteins and it exerts its cellular functions by actively transporting phospholipids across cellular membranes.^1^ The *ATP8B1* gene is expressed in liver and in several non-hepatic tissues, including the small and large intestine.^2^ Mutations in *ATP8B1* lead to progressive familial intrahepatic cholestasis 1 [PFIC1], a chronic autosomal recessive liver disorder, which is characterized by impaired bile formation and progressive liver damage culminating in fibrosis and cirrhosis.^3^ Patients usually present in the first year of life with jaundice and pruritis.^4,5^ PFIC1 patients also present extrahepatic phenotypes including intestinal malabsorption, failure to thrive, diarrhoea and hearing-loss.^5–8^ Liver transplantation is as yet the only life-saving resource, which relieves the cholestasis, but extrahepatic manifestations, including gastrointestinal dysfunction, remain a major complication as diarrhoea often persists or even worsens.^9–11^ The reasons for the intestinal problems are incompletely understood but have been associated with impaired localization of apical membrane proteins, including alkaline phosphatase, sucrase isomaltase, ASBT and CFTR, and an affected electrolyte balance in faecal water of PFIC1 patients.^12–14^ The restored bile flow in post-liver transplant PFIC1 patients causes re-entry of high concentrations of bile salts into the intestine, which consequently aggravates the intestinal phenotype.^15^ Recently, two groups independently reported inflammation in the intestine of PFIC1 patients who had undergone a liver transplantation.^10,16^ The consequence of ATP8B1 dysfunction on intestinal homeostasis and inflammation is as yet unclear. Therefore, here we investigated the role of ATP8B1 in the intestine *in vivo* and *in vitro* as well as in relation to inflammatory bowel disease [IBD].

In this study, we show that ATP8B1 is highly expressed in epithelial cells of the intestine and is specifically reduced in patients with ulcerative colitis [UC] and in dextran sodium sulphate [DSS]-induced colitis in mice. We experimentally address the function of ATP8B1 in intestinal homeostasis by treating ATP8B1-deficient mice with DSS. ATP8B1-deficient mice displayed increased DSS sensitivity that was associated with increased intestinal permeability. Mechanistic studies in Caco2-BBE cells demonstrated an essential function for ATP8B1 in the establishment of epithelial barrier function.

## 2. Material and Methods

### 2.1. Patient material

Fresh tissue samples were taken from IBD patients who underwent colorectal resection in the Amsterdam University Medical Center, location AMC. Normal colonic samples were taken from colorectal cancer patients at least 5 cm from the tumour. The study protocol was approved by the Medical Ethical Committee of the AMC [IBD Biobank, METC2014-178] and all patients provided written informed consent. Samples were immediately snap frozen and stored at −70°C until extraction, or fixed in 4% formaldehyde and routinely embedded in paraffin. Paraffin-embedded colorectal biopsies were obtained from a PFIC1 patient after liver transplantation that has been described by Kavallar and colleagues under IRB approval of the Medical University of Innsbruck [EK 1029/2017].^17^

### 2.2. Analysis of transcriptome data sets

Whole-transcriptome data were derived from Gene Expression Omnibus [GEO] dataset GEO: GSE75214 (healthy control [HC], Crohn’s disease [CD] and UC), GSE16879 [HC, matched pre-therapeutic and post-therapeutic biopsies of infliximab-treated UC and CD], GSE38713 [HC and UC], GSE109142 [HC and UC], and GSE10616 [HC and UC]; and mouse: GSE42768 [DSS-colitis], GSE12223 [IL10KO], and GSE27302 [T-cell transfer]. The above-mentioned data sets were uploaded to and analysed by the R2: Genomics Analysis and Visualization Platform [http://r2.amc.nl].

### 2.3. Mice

All experiments were approved by the Dutch government [Centale Commissie Dierproeven] under approval no. AVD1180020185344, and performed in accordance with national guidelines and regulations. *Atp8b1*^*G308V/G308V*^/c57Bl/J6 and wild-type c57Bl/J6 mice were bred in house and mice 6–10 weeks old of both genders were used, equally distributed over the groups. Mice were kept under specific pathogen-free conditions in individually ventilated cages. For induction of colitis drinking water was supplemented with 2.0% [w/v] DSS [mol. wt 36 000–50 000 kDa; Sigma Aldrich], provided *ad libitum* and refreshed every other day. Body weights were measured daily during DSS administration. Mice were killed by CO_2_ suffocation and the large intestine was removed. All measurements were performed in a blinded fashion [see Supplementary Material & Methods]. To determine intestinal permeability *in vivo*, mice were starved for 4 h after which FITC-conjugated dextran [4 kDa, 4FD; Sigma-Aldrich] was administered by oral gavage [60 mg/100 g body weight]. After 3 h, mice were killed and blood was collected by cardiac puncture. Plasma samples were measured for FITC fluorescence intensity [ex/em = 485/520 nm] using a CLARIOstar monochromator microplate reader [BMG Labtech].

### 2.4. Mice colitis severity scores

The Disease Activity Index [DAI, score 0–9] was determined, consisting of severity of oedema [0–3], diarrhoea [0–3], and faecal occult blood [0–3]. The length of the large intestine was measured in a relaxed position without stretching and weighed after removal of faeces. Colon density [mg/cm] was calculated by dividing the weight by the length of the large intestine. The intestine was opened longitudinally and washed thoroughly with phosphate-buffered saline [PBS], longitudinally divided in two, and one side was processed as a ‘swiss role’ into paraffin, the other side being immediately snap frozen and stored at −70°C. Slides of 5 µm were cut and stained with haematoxylin and eosin [H&E] using a standard protocol, and a standardized scoring system [score 0–12] was used to assess the severity of colitis consisting of two parameters: severity of inflammation [0–6]: 0 = none; 1 = infiltration in the lamina propria; 2 = confluence of inflammatory cells extending into the submucosa; 3 = transmural extension of the inflammatory infiltrate, combined with the extent of the inflammation [0 = none; 1 = <25%; 2 = 25–50%; 3 = >50%], and crypt damage [0–6]: 0 = intact crypts; 1 = loss of the basal half of the crypt; 2 = entire crypt loss; 3 = erosions/complete loss of epithelial layer, combined with extent [1 = <25%; 2 = 25–50%; 3 = >50%].

### 2.5. *In situ* hybridization

Sections of 5 µm were cut freshly and dried overnight at 37°C. *In situ* hybridization was performed according to the manufacturer’s guidelines using the RNAscope Reagent 2.5HD Brown system [Advanced Cell Diagnostics] using the *mmAtp8b1or hsATP8B1* probe [Bio-techne Ireland].

### 2.6. Immunohistochemistry

Sections of 5 µm were deparaffinized in xylene and rehydrated. Endogenous peroxidase was blocked using 0.3% H_2_O_2_ in methanol for 30 min. For ATP8B1 antigen retrieval slides were cooked at 100°C for 20 min in 0.01 M sodium citrate pH 6.0. For CLDN4 antigen retrieval slides were cooked at 100°C for 10 min in EDTA pH 9.0. After antigen retrieval slides were blocked in PBS with 1% bovine serum albumin [1% BSA/PBS] for 30 min at room temperature [RT], followed by incubation overnight at 4°C with a primary antibody: anti-human ATP8b1 [1:50, Thermo Fischer Scientific, PA5-53839] or CLDN4 [1:400, Invitrogen, 36-4800] in 1% BSA/PBS. Antibody binding was visualized using Powervision horseradish-peroxidase [HRP]-labelled secondary antibodies from Immunologic and diaminobenzidine [Sigma-Aldrich] for substrate development. All sections were counterstained with Mayer’s haematoxylin [Sigma-Aldrich].

### 2.7. Intestinal tissue homogenates

Intestinal tissues were homogenized in cell lysis buffer [Cell Signaling Technology] with protease inhibitors [Roche] using Precellys tissue homogenizer tubes [Bertin Technologies] for four times for 15 min at 5000 r.p.m. at 4°C. Samples were then spun down for 10 min at 14 000 r.p.m. at 4°C and the supernatant was transferred to a clean tube. Protein concentrations were determined via a BCA kit [ThermoFischer Scientific].

### 2.8. Immunoblotting

Samples were run on SDS-PAGE gels under reducing conditions and transferred to nitrocellulose membranes [GE Health Care]. Membranes were blocked by incubation in 5% non-fat milk [Nutricia] in TBST [TBS + 0.1% Tween-20] for 2 h at RT and subsequently incubated with antibodies: ATP8B1,^18^ Claudin-2 [clone E1H9O, CST], Claudin-3 [Invitrogen, 34-1700], Claudin-4 [Invitrogen, 36-4800], JAM-A [Clone EP1042Y, Abcam], Occludin [Proteintech, 13409-1-AP], and Villin [Santa Cruz, sc-7672] in 2% milk/TBST overnight at 4°C. After incubation, membranes were washed three times with TBST, incubated with HRP-conjugated secondary antibodies [Dakocytomation] in 2% milk/PBST for 2 h at RT. Expression was detected by Lumilight Plus [Roche]. Blots were then stripped in stripping buffer [ThermoFischer Scientific] for 10 min at RT and incubated with GAPDH antibody [CST, 14C10] or β-Actin antibody [Sigma, AC15]. Optical density [OD] was determined using ImageJ software [ImageJ 1.50i, W. Rasband, National Institutes of Health; http://imagej.nih.gov/ij/].

### 2.9. Cell culture

Caco2-BBE cells were obtained from the ATCC and cultured in DMEM [Dulbecco’s modified Eagle medium; Lonza], 10% fetal calf serum [FCS], 1% penicillin/streptomycin [Invitrogen] and 5 mM glutamine [Lonza]. Caco2-BBE cells were seeded at a density of 3.0 × 10^4^ cells/100 µL in transwell culture inserts [6.5 mm, 0.4 µm pore size, VWR] and cultured up to 21 days. For short cultures, Caco2-BBE cells were seeded at a density of 5 × 10^4^ cells/100 µL per transwell.

### 2.10. TEER and 4FD permeability assays

Transepithelial electrical resistance [TEER] was measured with a Millicell ERS-2 Voltohmeter [Millipore], according to the manufacturer’s instructions. TEER values were corrected for background values [empty transwell] and surface area [0.33 cm^2^]. To evaluate the paracellular permeability of the Caco2-BBE monolayers, translocation of 4FD [Sigma-Aldrich] from the apical to basolateral side was measured. Therefore, 100 µL of 1 mg/mL 4FD in culture medium was added to the apical chamber and the amount of 4FD in the basolateral compartment was determined after 2 h using a CLARIOstar monochromator microplate reader [BMG Labtech].

### 2.11. Calcium switch assay

To study tight junction [TJ] assembly, TJs were disrupted by the Ca^2+^ chelator EGTA [Sigma-Aldrich]. Transwell monolayers cultured for 21 days were washed twice with calcium-free, magnesium-free EBSS [Eagle’s balanced salt solution], before treatment with 2 mM EGTA in EBSS [both basolateral and apical] for 20 min. The monolayers were then washed once with EBSS and once with culture medium, and replaced by culture medium. TEER was measured before calcium-switch, immediately after and then every 2 h.

### 2.12. Lentivirus transduction

Knockdown cell lines for ATP8B1 and CLDN4 were generated by lentiviral transduction using short-hairpin RNA [shRNA] vectors to *ATP8B1* [TRCN0000050127] and *CLDN4* [TRCN0000116627], respectively, from the Mission shRNA Library [Sigma-Aldrich]. The non-targeting hairpin SHC002 in pLKO.1-puro [CAACAAGATGAAGAGCACCAA] was included as a control. Selection was performed using 10 µg/mL puromycin [Sigma-Aldrich]. The lentiviral vector to express enhanced green fluorescent protein [eGFP]-tagged ATP8B1 was described previously.^19^

### 2.13. Immunofluorescent staining of cells

Transwell monolayers or cells grown on glass cover slips were washed with PBS and fixed with 4% paraformaledehyde for 10 min at RT. After thorough washings with PBS, cells were permeabilized with 0.1% Triton in PBS [PBS/Tx] and blocked in 5% goat serum [Dako] in 1% BSA/PBS and subsequently incubated with antibody to Claudin-4 [1:100] in PBS/Tx for 2 h at RT and washed with PBS/Tx. Cells were then incubated with goat-anti-rabbit-alexaFluor488 or alexaFluor594 [Invitrogen] for 1 h at RT, washed with PBS/Tx, and mounted with ProLong Gold Antifade reagent with DAPI [Thermo Fisher Scientific]. Images were taken with a Leica DM6000 microscope using LAS AF software [Leica]. For transverse sections, transwells were fixed with 4% paraformaldehyde for 30 min at RT and washed with PBS twice. After storing in 70% ethanol, transwells were cut from the chambers and imbedded in histogel [Thermo Scientific], embedded in paraffin and sectioned. CLDN4 immunofluorescent staining was done after antigen retrieval using Tris-EDTA pH 9.0 in a co-staining with goat-anti-GFP [1:100, Rockland, 600-101-215] to amplify the eGFP signal, and donkey-anti-rabbit-AlexaFluor546 [1:500, Invitrogen, A10040] and donkey-anti-goat-AlexaFLuor488 as secondary antibodies.

### 2.14. Proliferation assays

Cell proliferation was measured using the CellTiter 96 AQueous One Solution Cell Proliferation Assay [Promega]. In total, 2 × 10^3^ cells were seeded in 100 µL per well in a 96-well plate and after 1, 2, 3, and 4 days 20 µL MTS solution was added and the absorbance was measured at 490 nm after 4 h. The absorbance values were corrected for the background [cell culture medium with MTS] values. Additionally, 1 × 10^3^ cells were plated in six-well plates and cultured for up to 2 weeks. Cells were then fixed with 4% formaldehyde in PBS for 15 min and subsequently stained with 5 mg/mL crystal violet [C3886, Sigma-Aldrich] in 2% ethanol for 20 min. After thorough washings with H_2_O plates were air-dried and photographed. Subsequently stained cells were lysed in 200 µL lysis buffer [CST], 100 µL was transferred to a 96-well plate, and crystal violet absorbance was measured at 570 nm and corrected for blank [lysis buffer only] values.

### 2.15. Quantitative reverse-transcription PCR

RNA isolation and FACSorting of human and mouse colonic cell populations was as described previously.^20^ For cDNA synthesis equal concentrations of mRNA were reverse transcribed using oligo[dT], random hexamers, and M-MuLV reverse transcriptase [RT] from the First Strand cDNA Synthesis kit [Thermo Scientific] according to the manufacturer’s protocol. Quantitative RT-PCR [qPCR] was performed using a sensifast SYBR No-ROX Kit [Bioline] on a BioRad iCycler. GeneNorm was used to select multiple stable housekeeping genes. Analysis was performed by using the LinReg method.^21^ Primer sequences to human *ATP8B1*: forward 5ʹ-TGGTGGATAGGACTGATGGTC-3ʹ, reverse 5ʹ-CGTTTACCAGGGCACCTTC-3ʹ and mouse *Atp8b1*: forward 5ʹ- GTCTGGGACAGAGTCATTTC-3ʹ and reverse 5ʹ-CTTATCAGAGAAGATGTAATG-3ʹ.

### 2.16. Statistical analysis

Data were analysed using a Kruskal–Wallis test followed by Dunn’s post-hoc test, analysis of variance [ANOVA] followed by Sidak’s post-hoc, Mann–Whitney test, unpaired t-test, or Wilcoxon signed rank test using Graphpad Prism 7.02 [Graphpad Software]. Data are presented as bar-scatterplots with bars indicating mean and dots representing individual values/animals or as bar graphs representing mean + SEM. Values of *p* < 0.05 were considered significant with *p < 0.05, **p < 0.01, ***p < 0.001, and ****p < 0.0001.

## 3. Results

### 3.1. ATP8B1 is downregulated in UC

**Figure 1.**
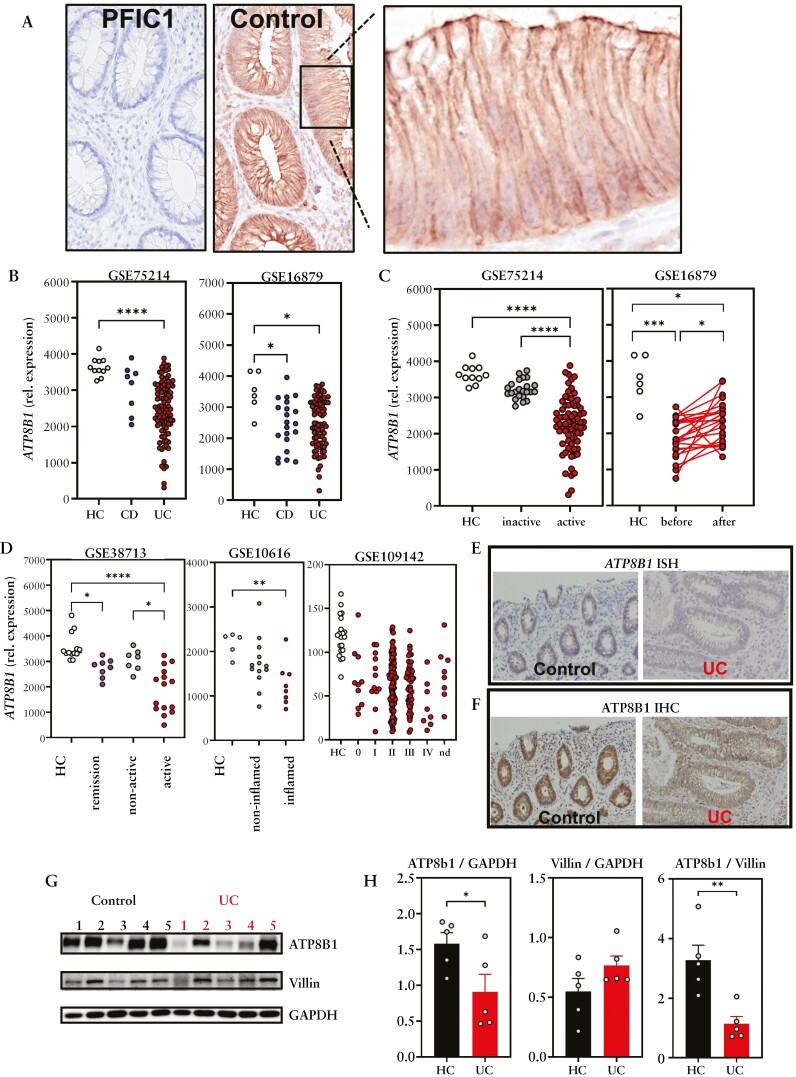
ATP8B1 expression in UC. [A] ATP8B1 expression in the human intestine as determined by immunohistochemistry on colonic tissue of a healthy control [HC] and a ATPB1-deficient PFIC1 patient. [B] Analysis of human *ATP8B1* expression in HC and UC and CD colonic biopsies. Data were derived from GSE75214 [HC, *n* = 11; CD, *n* = 8; UC, *n* = 97] and GSE16879 [HC, *n* = 6; CD, *n* = 37; UC, *n* = 78]. [C] *ATP8B1* expression in active [*n* = 74] vs inactive UC [*n* = 23], and before [*n* = 24] vs after [*n* = 24] infliximab treatment. [D] Analysis of *ATP8B1* expression in GSE38713 [HC, *n* = 13; UC remission, *n* = 8; non-active UC, *n* = 7; active UC, *n* = 15], GSE10616 [HC, *n* = 5; non-inflamed UC, *n* = 13; inflamed UC, *n* = 8], and GSE109142 (HC, *n* = 20; UC, histological severity 0, *n* = 10; I, *n* = 12; II, *n* = 116; III, *n* = 52; IV, *n* = 8; not determined [ND], *n* = 8). [E] *ATP8B1 in situ* hybridization and [F] ATP8B1 immunohistochemical staining in control and active UC [representative images from *n* = 3 patients]. [G] ATP8B1, Villin, and GAPDH immunoblot in UC vs HC [both *n* = 5], quantified in H. Data are presented as scatterplots representing individual values or bar graphs representing mean + SEM. Data were analysed using a Kruskal–Wallis test followed by Dunn’s post-hoc test in B, C, and D, and an unpaired t-test in G: *p < 0.05, **p < 0.01, ***p < 0.001, and ****p < 0.0001.

To investigate the expression of *ATP8B1* in the human intestine we first analysed publicly available gene expression data sets, including ileal and colonic tissue samples from healthy control individuals. This revealed that *ATP8B1* is highly expressed in the colon, when compared to the ileum [Supplementary Figure S1A]. Analysis of single cell RNA sequencing [scRNAseq] data indicated that *ATP8B1* is highly expressed by epithelial cells and endothelial cells in both the small intestine and colon [Supplementary Figure S1B]. This was corroborated by qPCR on cell sorted populations from human colonic tissue [Supplementary Figure S1C]. Immunohistochemistry indicated that ATP8B1 protein is mainly detected in the apical domain, as well as in lateral membranes of colonic intestinal epithelial cells, and confirmed complete absence of ATP8B1 protein in a colorectal biopsy of a PFIC1 patient [Figure 1A]. Notably, the colorectal biopsy from the PFIC1 patient showed overt signs of chronic inflammation [Supplementary Figure S1D]. Next, we sought to interrogate whether *ATP8B1* expression was changed within the gastrointestinal tract in inflammatory conditions. Therefore, we first analysed published transcriptomic data of intestinal biopsies from two different cohorts of IBD patients [GSE16879, GSE75214], including CD and UC. As *ATP8B1* expression is highest in the colon, we focused on the colonic expression as this also enabled us to compare CD with UC. *ATP8B1* expression was significantly decreased in both UC patient cohorts, while only one cohort showed a significant decrease of *ATP8B1* expression in CD [Figure 1B]. Further analysis of both cohorts showed that decreased *ATP8B1* expression was specifically associated with active UC when compared to non-active UC [Figure 1C]. In line with this, *ATP8B1* was increased upon disease treatment [Figure 1C, ‘before versus after’]. Analysis of additional patient cohorts confirmed that *ATP8B1* expression was decreased in diseased intestinal tissue of UC patients [Figure 1D]. *In situ* hybridization as well as immunohistochemical staining showed decreased ATP8B1 levels in UC compared to healthy colon tissue [Figure 1E]. Immunoblotting of intestinal colon samples indeed confirmed ATP8B1 protein levels being reduced in UC compared to healthy controls [Figure 1F, G]. Villin levels were not affected in UC samples, indicating that the decreased ATP8B1 levels were not simply a reflection of loss of epithelial cells during ulcerating conditions [Figure 1F, G].

### 3.2. Atp8b1 expression in experimental mouse models of colitis

**Figure 2.**
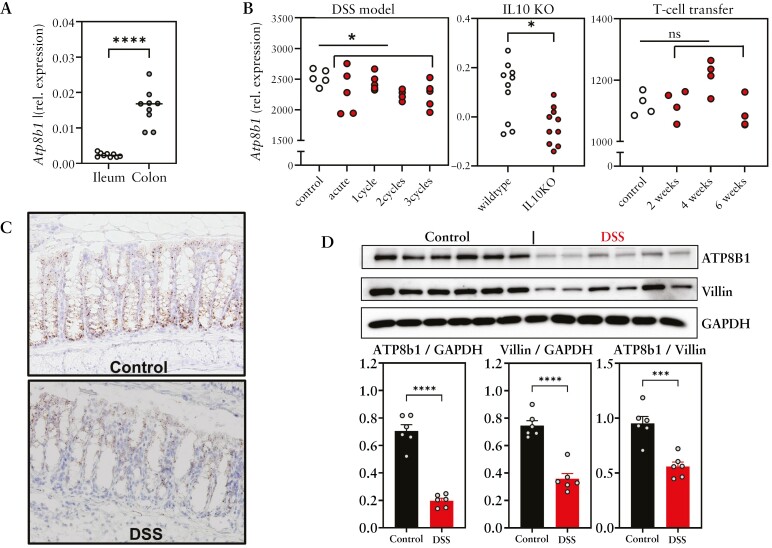
ATP8B1 expression in mouse IBD models. [A] *Atp8b1* expression in the mouse intestine as determined by qPCR. [B] Analysis of *Atp8b1* expression in the DSS model [GSE42678], IL10KO [GSE12223], and T-cell transfer [GSE27302]. [C] *Atp8b1 in situ* hybridization in acute DSS colitis. [D] ATP8B1 and GAPDH immunoblot in acute DSS vs control colons [both *n* = 6], quantified in E. Data are presented as scatterplots representing individual values or bar graphs representing mean + SEM. Data were analysed using Mann–Whitney tests in A and B and an unpaired t-test in D: *p < 0.05, **p < 0.01, ***p < 0.001, and ****p < 0.0001.

Next, we investigated intestinal expression of *Atp8b1* in several murine models of IBD. Similar to humans, mice expressed higher levels of *Atp8b1* in the colon compared to ileum [Figure 2A]. scRNAseq revealed that in mice *Atp8b1* is highly expressed in epithelial cells, but also in fibroblasts and cells of the lymphatic system [Supplementary Figure S2A, B]. Upon induction of intestinal inflammation with DSS, an experimental IBD model that resembles UC, a decrease in *Atp8b1* expression was detected [Figure 2B]. Additionally, in IL10 knock-out mice, that spontaneously develop [UC-like] intestinal inflammation, a reduction in intestinal *Atp8b1* expression was overt [Figure 2B]. In contrast, in intestinal inflammation of the T-cell transfer model, which is considered more CD-like, intestinal *Atp8b1* expression was unaffected [Figure 2B]. *In situ* hybridization confirmed the reduction of epithelial *Atp8b1* in DSS-induced colonic inflammation [Figure 2C] and this coincided with reduced ATP8B1 protein levels [Figure 2D], also when corrected for epithelial cell abundance by Villin levels [Figure 2D].

### 3.3. DSS induces severe colitis in ATP8B1-deficient mice

**Figure 3.**
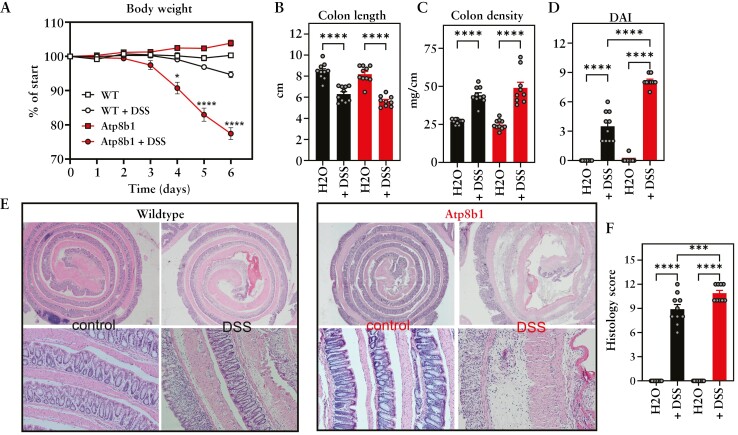
Severe DSS colitis in the *Atp8b1* mouse. [A] Body weight of *Atp8b1* and wild-type mice treated with 2% DSS or normal drinking water [all groups, *n* = 10]. [B] Colon length, [C] colon density, [D] DAI, and [E] representative histological images, quantified for colitis severity in F. Data are presented as scatterplots representing individual values or bar graphs representing mean + SEM. Data were analysed using ANOVA followed by Sidak’s multiple comparison tests: *p < 0.05, **p < 0.01, and **** *p* < 0.0001. In A significance of *Atp8b1* + DSS vs WT + DSS is shown.

To examine if loss of ATP8B1 in the intestine affects colonic inflammation, we challenged *Atp8b1*^*G308V/G308V*^ mutant mice with DSS. *Atp8b1*^*G308V*/*G308V*^ mutant mice [hereafter denoted *Atp8b1*-deficient mice] are knock-in mice for the prototypic PFIC1 mutation from the Byler family,^3^ a glycine to valine substitution at position 308 [G308V], that results in near absence of the protein.^22^*Atp8b1*-deficient mice show an exacerbated response to DSS compared to wild-type control mice, reflected by a >20% decrease in body weight loss 6 days post-DSS [Figure 3A]. Similar to wild-type mice on DSS, colon length [Figure 3B] and colon density [Figure 3C] were decreased and increased, respectively, indicating colonic inflammation. Compared to DSS-treated wild-type mice, *Atp8b1*-deficient mice displayed an increased DAI [Figure 3D] and histopathology score, as evidenced by a thickening of the sub-mucosa, massive immune cell infiltration, and epithelial membrane damage [Figure 3E, F]. These experiments show that ATP8B1 plays a crucial role in the intestine in DSS-induced colitis.

### 3.4. *ATP8B1* is involved in intestinal epithelial barrier function

**Figure 4.**
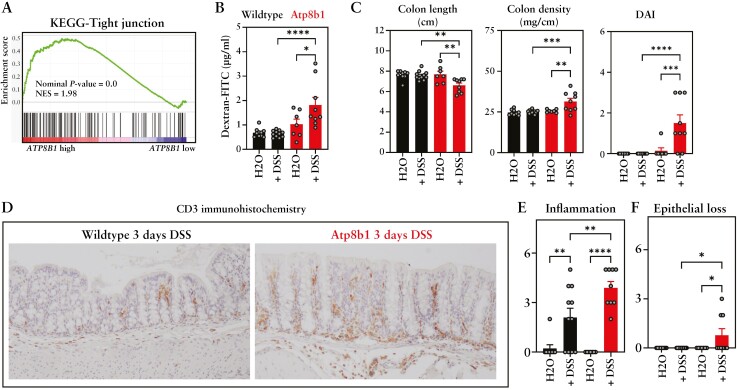
ATP8B1 is important in epithelial barrier function. [A] Enrichment plot of the tight junction pathway of UC samples with high vs low *ATP8B1* expression, based on median *ATP8B1* expression [GSE109142; both groups *n* = 103]; NES = normalized enrichment score. [B] *In vivo* intestinal barrier permeability as measured by 4FD in plasma. [C] Colon length, colon density, and DAI in *Atp8b1* (in red; control [*n* = 7] vs DSS [*n* = 9]) and in wild-type mice (in black; control [*n* = 10] vs DSS [*n* = 11]) under homeostatic conditions and after 3 days of 2% DSS challenge. [D] Representative CD3 immunohistochemistry of colon sections from *Atp8b1* and wild-type mice treated for 3 days with DSS. [E] Inflammation and [F] crypt damage scores. Data were analysed using ANOVA followed by Sidak’s multiple comparison tests: *p < 0.05, **p < 0.01, *** *p* < 0.001, and **** *p* < 0.0001.

To identify molecular pathways that correlated with reduced intestinal *ATP8B1* expression in UC patients, we investigated gene expression associations in an unbiased manner by splitting intestinal UC samples into two groups according to their *ATP8B1* expression [i.e. above and below the median ATP8B1 expression] in a patient cohort [GSE109142, *n* = 206]. Gene set enrichment analysis [GSEA] revealed a close correlation between TJ pathway-associated gene expressions and *ATP8B1* expression [Figure 4A], suggesting that ATP8B1 deficiency is associated with a defect in intestinal barrier function.

To investigate whether enhanced sensitivity to DSS colitis in *Atp8b1*-deficient mice is associated with a diminished epithelial barrier function, *Atp8b1*-deficient mice were challenged for 3 days with 2% DSS. This allowed us to assess the epithelial barrier integrity *in vivo* upon the initial epithelial insult, but before the manifestation of a severe colitis. After 3 days of DSS treatment there were no signs of body weight loss in either wild-type or *Atp8b1*-deficient mice [Supplementary Figure S3]. Intestinal permeability, as assessed after oral gavage of 4FD, was not affected in water- or DSS-treated wild-type mice nor in water control *Atp8b1*-deficient mice, indicating that barrier function was not impaired 3 days post-DSS, nor in naïve *Atp8b1*-deficient mice [Figure 4B]. Importantly, intestinal passage of 4FD was significantly increased in DSS-treated *Atp8b1* mice [Figure 4B], indicating a decreased epithelial barrier even 3 days after a DSS-induced epithelial insult. At this time-point, DSS-treated *Atp8b1*-deficient mice showed clear macroscopic signs of intestinal inflammation, as indicated by a decreased colon length and increased colon density and DAI [Figure 4C]. Microscopically, mild intestinal infiltration of immune cells [e.g. CD3^+^ T-cells] was observed in wild-type mice that was aggravated in *Atp8b1*-deficient mice [Figure 4D, E]. The massive loss of epithelial cells, which is characteristic for the DSS model at later time-points [and observed 6 days post-DSS treatment; Figure 3E], had not yet occurred in most animals [Figure 4F]. These data indicate that ATP8B1 function is essential to preserve a tight intestinal barrier in the initiating events of DSS-induced colitis.

### 3.5. ATP8B1 is involved in establishment of the epithelial barrier *in vitro*

**Figure 5.**
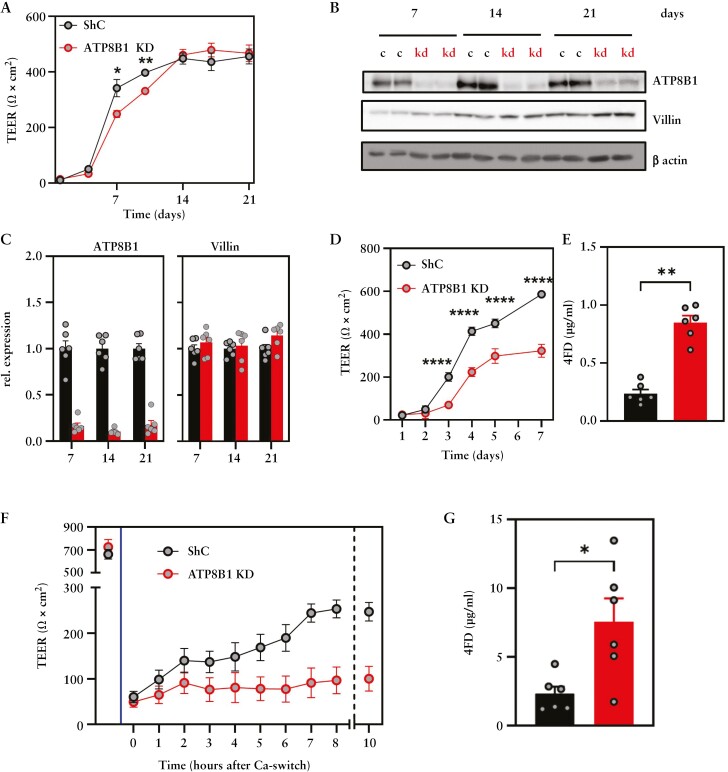
ATP8B1 has a role in epithelial barrier establishment in Caco2-BBE cells *in vitro.* [A] TEER measurements of a long-term [21 day] culture of shControl and ATP8B1 KD Caco2-BBE cells. [B] ATP8B1, Villin, and β-actin immunoblot at several time-points during Caco2-BBE cell differentiation, quantified in C and expressed relative to shControl cells per time-point; see blots in Supplementary Figure S4. [D] TEER measurements over a 7-day culture of shControl and ATP8B1 KD Caco2-BBE cells with [E] quantification of 4FD passage at day 7. [F] TEER measurements before and after calcium withdrawal and after re-establishment of the epithelial barrier in shControl and ATP8B1 KD Caco2-BBE cells with [G] quantification of 4FD passage 10 h after the Ca switch. Vertical dotted line at *t* = 9 h indicates refreshment of medium. Black: shControl cells; red: ATP8B1 KD cells. Representative of two to four independent experiments. Data were analysed using unpaired t-tests: *p < 0.05, **p < 0.01, ***p < 0.001 and ****p < 0.0001.

Next, we investigated the contribution of ATP8B1 to epithelial barrier function in more detail in Caco2-BBE cells, which when grown on transwell filters differentiate and establish an effective epithelial barrier as characterized by a gradual increase in TEER over a ~21-day culture period and concomitant elevation of epithelial differentiation markers, including Villin [Figure 5A]. Similarly, ATP8B1 levels increased with increased differentiation status of the cells [Figure 5B], suggesting a relationship between ATP8B1 expression and epithelial barrier formation. Although there was no difference in TEER values in fully differentiated cells, ATP8B1 knockdown [KD] Caco2-BBE cells exhibited a somewhat delayed development of TEER over the first 14-day culture period [Figure 5]. The delayed TEER development was not caused by impaired differentiation or proliferation as neither Villin levels nor cellular proliferation rates were affected [Figure 5B, C and Supplementary Figures S4 and S5].

To accelerate attaining confluence and barrier function, we seeded a higher cell density in the transwell filters. Under these conditions, and as reported by others, the TJs are assembled within 4 days.^23^ Notably, during the first 7 days, TEER values of ATP8B1 KD cells lagged behind control cells by ~50% [Figure 5D], a phenotype that coincided with a 4-fold increased passage of 4FD [Figure 5E]. Despite the lag in initial TEER development, resistance was completely recovered after 21 days of culture, suggesting a role for ATP8B1 in the initial phase of barrier establishment. We therefore performed a calcium switch assay, where we disrupted the 21-day fully differentiated TJ complexes after which we monitored restoration of barrier function. Ca^2+^ withdrawal resulted in an ~85% drop in TEER, which was equal in both cell lines [Figure 5F and Supplementary Figure S6]. After Ca^2+^ re-addition, ATP8B1 KD cells displayed a significantly reduced recovery of TEER, suggesting a delayed barrier reassembly [Figure 5F]. The delayed development of TEER coincided with an ~3-fold increase in permeability to 4FD relative to short-hairpin control (shControl) cells [Figure 5G]. Altogether, these data indicate a crucial role for ATP8B1 in the initial establishment of the epithelial barrier.

### 3.6. ATP8B1 and Claudin-4 have complementary functions in barrier function in Caco-2 cells

**Figure 6.**
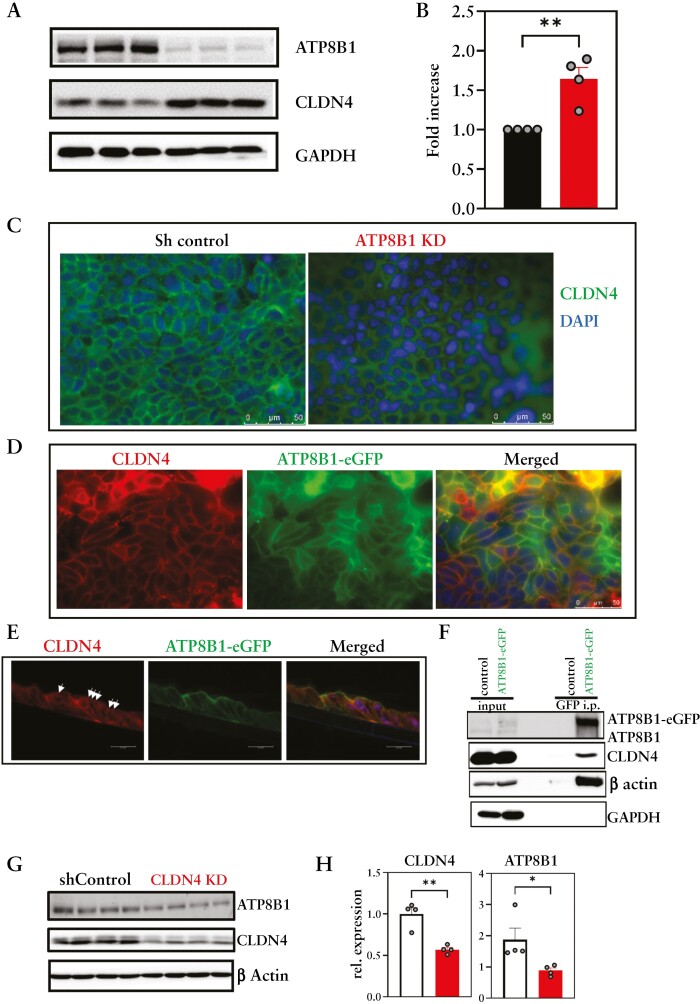
ATP8B1 and CLDN4 interact in Caco2-BBE cells. [A] Representative immunoblot analysis of ATP8B1, CLDN4, and GAPDH in ATP8B1 KD cells, of which CLDN4 levels were quantified from four independent experiments [see Supplementary Figure S7] shown in B. [C] Immunofluorescence detection of CLDN4 in 7-day transwell cultures of Caco2-BBE shControl and ATP8B1 KD cells. [D] Immunofluorescence detection of CLDN4 in ATP8B1-eGFP-expressing Caco2-BBE cells. [E] Immunohistochemical staining of ATP8B1-eGFP and CLDN4 on translateral sections of ATP8B1-eGFP-expressing Caco2-BBE cells grown on transwell inserts for 7 days. [F] Anti-GFP co-immunoprecipitation of ATP8B1-eGFP showing co-immunoprecipitation of CLDN4 and β-actin, but not of GAPDH. [G] Immunoblot analysis of ATP8B1, CLDN4, and β-actin in CLDN4 KD Caco2-BBE cells, quantified in H. All data shown are representative of two or three independent experiments. Data were analysed using unpaired t-tests: *p < 0.05, **p < 0.01, ***p < 0.001, and ****p < 0.0001.

To investigate the molecular mechanisms involved in ATP8B1-associated barrier establishment, we assessed the levels of several TJ proteins on day 7 transwell inserts. ATP8B1 KD cells displayed no/little changes in multiple TJ protein levels, except for CLDN4 levels, which were slightly elevated [Figure 6A, B, Supplementary Figure S7]. Since elevated CLDN4 levels have been associated with increased rather than impaired barrier function,^24^ we investigated the cellular localization of CLDN4 in the ATP8B1 KD monolayers. Immunofluorescence staining revealed that ATP8B1 KD cells showed a diffuse cytoplasmic staining pattern for CLDN4, as opposed to a membrane-associated CLDN4 staining in control cells [Figure 6C]. These data suggest that in ATP8B1 KD cells, CLDN4 is not properly localized and as a consequence accumulated in these cells.

**Figure 7.**
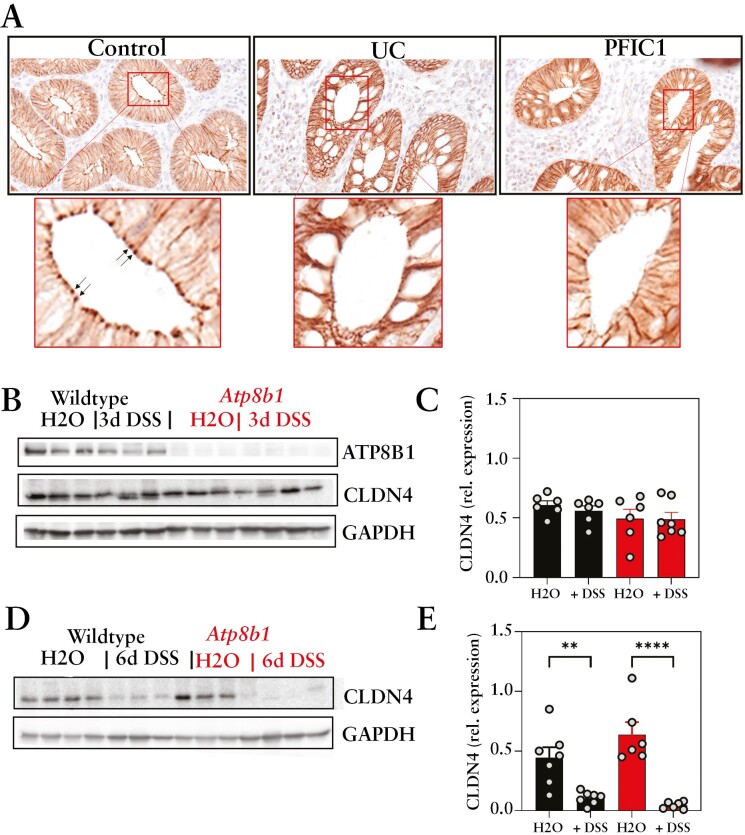
CLDN4 and ATP8B1 in UC and DSS colitis. [A] Immunohistochemical staining of CDLN4 in colonic tissue of healthy controls and [active] UC patients [representative images from *n* = 3 patients] and an ATPBB1-deficient [PFIC1] patient. [B–E] ATP8B1, CLDN4, and GAPDH immunoblots of colon homogenates of water-control and 3-day [B] or 6-day [D] DSS-treated wild-type and *Atp8b1* mice, quantified in C [wt control, *n* = 6; wt + DSS, *n* = 6; *Atp8b1* control, *n* = 6; *Atp8b1 *+ DSS, *n* = 7] and E [wt control, *n* = 7; wt + DSS, *n* = 7; *Atp8b1* control, *n* = 6; *Atp8b1 *+ DSS, *n* = 7], respectively. Data in C and E were analysed using ANOVA followed by Sidak’s multiple comparison tests: *p < 0.05, **p < 0.01, ***p < 0.001, and ****p < 0.0001.

To assess a possible interaction between ATP8B1 and CLDN4 we studied their localization in ATP8B1-eGFP over-expressing Caco2-BBE cells. The ATP8B1-eGFP fusion is functional,^19^ and its over-expression did not influence epithelial barrier function, as judged by TEER and 4FD permeability measurements [Supplementary Figure S8]. In Caco2-BBE cells grown in a monolayer ATP8B1-eGFP, as previously shown,^19^ localized to the plasma membrane and co-stained with CLDN4 [Figure 6D], suggesting that both proteins localized to or near the plasma membrane. Translateral slides of Caco2-BBE-ATP8B1-eGFP cells grown on transwells for 7 days confirmed apical membrane staining of ATP8B1-eGFP and showed a punctate enrichment for CLDN4, reminiscent of tight junctional staining [Figure 6E]. To assess whether the two proteins could physically interact, we co-immunoprecipitated ATP8B1-eGFP from the cells and eluted a small fraction of CLDN4, suggesting that ATP8B1 could interact with CLDN4 [Figure 6F]; this interaction was specific for CLDN4 since ATP8B1-eGFP did not precipitate with GAPDH. Furthermore, ATP8B1 was clearly attached to the cytoskeleleton, indicated by β-actin co-immunoprecipitation. These data suggest strongly that a small fraction of the cellular CLDN4 pool specifically interacts with ATP8B1. To establish the contribution of CLDN4 to barrier formation, we generated CLDN4 KD Caco2-BBE cells. Knockdown led to a strong decrease in cellular proliferation [Supplementary Figure S9] which hampered measurement of the epithelial barrier by TEER and 4FD translocation. Immunoblot analyses showed that in CLDN4 KD cells, ATP8B1 levels were reduced [Figure 6G, H]. Although we cannot exclude a secondary consequence of affected proliferation, these data suggest an inter-dependence of the two proteins. Similar to control Caco2-BBE cells, immunohistochemical staining of CLDN4 in human healthy control colon showed clear tight junctional, as well as lateral, staining in epithelial cells [Figure 7C]. We found that tight junctional staining of CLDN4 was less pronounced or even absent in intestinal samples from UC patients whereas lateral staining was unaffected [Figure 7A]. This correlated with decreased ATP8B1 protein staining in the same UC samples [Figure 1A, E]. Importantly, the colonic epithelium of the ATP8B1-deficient [PFIC1] patient virtually lacked tight junctional signal for CLDN4 [Figure 7A].

In *Atp8b1*-deficient mice, both under control conditions and after 3 days of DSS exposure, intestinal CLDN4 levels were similar to those in wild-type mice [Figure 7B, C], which decreased dramatically after 6 days of DSS exposure in both wild-type and *Apt8b1*-deficient mice [Figure 7D, E]. Altogether, these data underline an important role for ATP8B1 in conjunction with CLDN4 in tight junctional integrity in intestinal epithelial cells.

## 4. Discussion

In the present study we provide evidence for an important role for the phospholipid flippase ATP8B1 in the pathogenesis of UC via the establishment of barrier function in intestinal epithelial cells. First, we show that *ATP8B1* was highly expressed in colonic intestinal epithelial cells and was down-regulated in colon tissue of patients with UC. Similarly, we show down-regulation of colonic *Atp8b1* in two mouse models of UC-like IBD. Second, *Atp8b1*-deficient mice were extremely sensitive to DSS exposure as indicated by the induction of wasting disease 6 days post-DSS, while wild-type mice showed only symptoms of mild disease. Third, we show that ATP8B1 fulfils a function in the establishment of the epithelial barrier both *in vivo* and *in vitro*. Furthermore, we show that ATP8B1 interacted with a small fraction of the cellular CLDN4 pool in Caco2-BBE cells. Importantly, CLDN4 was mislocalized in a colorectal biopsy of an ATP8B1-deficient [PFIC1] patient, while both ATP8B1 and CLDN4 were down-regulated and/or mislocalized in UC samples. Altogether, these observations suggest strongly that down-regulation of ATP8B1 can be an initiating trigger to impair intestinal barrier function, possibly via impairment of TJ integrity, with consequent aggravation of intestinal inflammation. Although our data show that barrier dysfunction in ATP8B1 low/deficient conditions is associated with CLDN4 mistargeting, we cannot rule out a possible contribution of other TJ proteins in this phenotype.

Our data indicate that ATP8B1 is crucially involved in the establishment, rather than in the homeostatic maintenance, of the intestinal epithelial barrier and that a ‘second hit’ is required to expose or aggravate an intestinal phenotype. This is evidenced by two observations *in vivo* and *in vitro*. First, *Atp8b1*-deficient mice do not display an impaired intestinal epithelial barrier and lack an overt intestinal phenotype. During a DSS-induced intestinal insult [‘second hit’], however, barrier dysfunction was observed even at 3 days post-DSS in *Atp8b1*-deficient mice, but not in wild-type mice, which progressed to severe epithelial damage and aggravating colitis 6 days after DSS. Second, ATP8B1 KD Caco-2-BBE cells displayed delayed barrier formation in the first 14 days of differentiation in a transwell culture, whereas barrier function was completely restored to that of control cells in fully differentiated cells. However, when a ‘second hit’ was employed on fully differentiated cells, i.e. barrier disruption by calcium withdrawal, re-establishment of the barrier was strongly impaired, indicating a role for ATP8B1 in barrier establishment.

The ‘second hit’ hypothesis could also explain the intestinal phenotype in post-liver transplant [LT] ATP8B1-deficient PFIC1 patients. Apart from severe cholestatic liver disease, extrahepatic phenotypes, including diarrhoea, are common in PFIC1 patients.^4,7,9,25^ After liver transplantation, however, >80% of PFIC1 patients continue to develop exacerbated diarrhoea,^26–28^ which is probably due to restored bile flow into the intestine. Besides diarrhoea, >90% of post-LT PFIC1 patients rapidly develop hepatic steatosis/steatohepatitis, both of which are reversible upon interruption of the enterohepatic circulation.^9,17,26,29–31^ We propose that increased bile salt entry into the intestine of post-LT PFIC1 patients serves a ‘second hit’ that damages the intestinal epithelium, causing epithelial barrier defects and consequent diarrhoea and inflammation, and triggers hepatic steatosis. Importantly, Stephanie and colleagues reported a mild and chronic inflammation in intestinal biopsies from a post-LT PFIC1 patient,^16^ while Henkel and colleagues recently reported on a post-LT PFIC1 patient who had developed colitis specifically in the distal colon.^10^ Hence, our observations indicate an essential function for ATP8B1 in the establishment of the intestinal barrier, and suggest strongly that a ‘second hit’ is required to expose a barrier defect as well as colitis when ATP8B1 levels are reduced/absent.

We find that both ATP8B1 and CLDN4 are involved in the epithelial barrier phenotype in Caco2-BBE cells. Claudins are integral membrane proteins that localize to TJs of all epithelial and endothelial cells where they play essential roles in paracellular permeability.^32–34^ With their extracellular domains they form paracellular seals, while in most claudins the intracellular C-terminal domains interact with other, PDZ-domain containing TJ proteins, signalling molecules, as well as with the actin network.^32,35,36^ In Caco2-BBE cells, we find a strong link between ATP8B1 and CLDN4. First, ATP8B1 KD Caco2-BBE cells showed reduced TEER build-up during the first 7 days in a transwell culture as well as elevated total CLDN4 levels. Although previous research showed that transgenic over-expression of CLDN4 resulted in increased TEER in MDCK cells,^37^ we hypothesize that the elevated CLDN4 levels in ATP8B1 KD cells are a consequence of mis-targeting and consequent cellular accumulation of CLDN4. Second, ATP8B1-eGFP and CLDN4 co-stained in the plasma membrane and co-immunoprecipitated, indicating that they physically interact. Our co-immunoprecipitation data indicate that only a small fraction of total CLDN4 precipitated with ATP8B1, which can be caused by either a low-affinity interaction between the two proteins or that just a small fraction of the CLDN4 pool is bound to ATP8B1. Collectively, our observations indicate that ATP8B1 has an important role in the establishment of barrier function, possibly via the maintenance of TJ integrity, in which CLDN4 plays an important role.

From our data we hypothesize that ATP8B1 is involved in the targeting of CLDN4, and possibly other yet to be identified proteins, to the TJ. This is underlined by our observations that CLDN4 did not localize TJs, whereas it was preserved in lateral membrane domains, a staining pattern that was previously reported by several others.^38–40^ Although our finding that elevated CLDN4 levels in ATP8B1 KD cells contrasts with the reduced CLDN4 levels found in UC patients,^38,41,42^ it is possible that the initial mislocalization [as observed in Caco2-BBE cells] causes the protein to accumulate, whereas under progressive inflammatory conditions, proteins, including CLDN4, will be degraded, decreasing total CLDN4 levels.

We have identified ATP8B1 as an essential protein in intestinal barrier establishment, and a novel, possible early-stage, determinant of UC pathogenesis.

## Supplementary Data

Supplementary data are available online at *ECCO-JCC* online.

jjae024_suppl_Supplementary_Figures_S1

jjae024_suppl_Supplementary_Figures_S2

jjae024_suppl_Supplementary_Figures_S3

jjae024_suppl_Supplementary_Figures_S4

jjae024_suppl_Supplementary_Figures_S5

jjae024_suppl_Supplementary_Figures_S6

jjae024_suppl_Supplementary_Figures_S7

jjae024_suppl_Supplementary_Figures_S8

jjae024_suppl_Supplementary_Figures_S9

## Data Availability

The data underlying this article are available in the article and in its online supplementary material.
