# Supplementary figures and images for: The Phospholipid Flippase ATP8B1 is Involved in the Pathogenesis of Ulcerative Colitis via Establishment of Intestinal Barrier Function
Source: J Crohns Colitis. 2024 Feb 16;18(7):1134–46. doi: 10.1093/ecco-jcc/jjae024 (PMC11302967; doi:10.1093/ecco-jcc/jjae024)

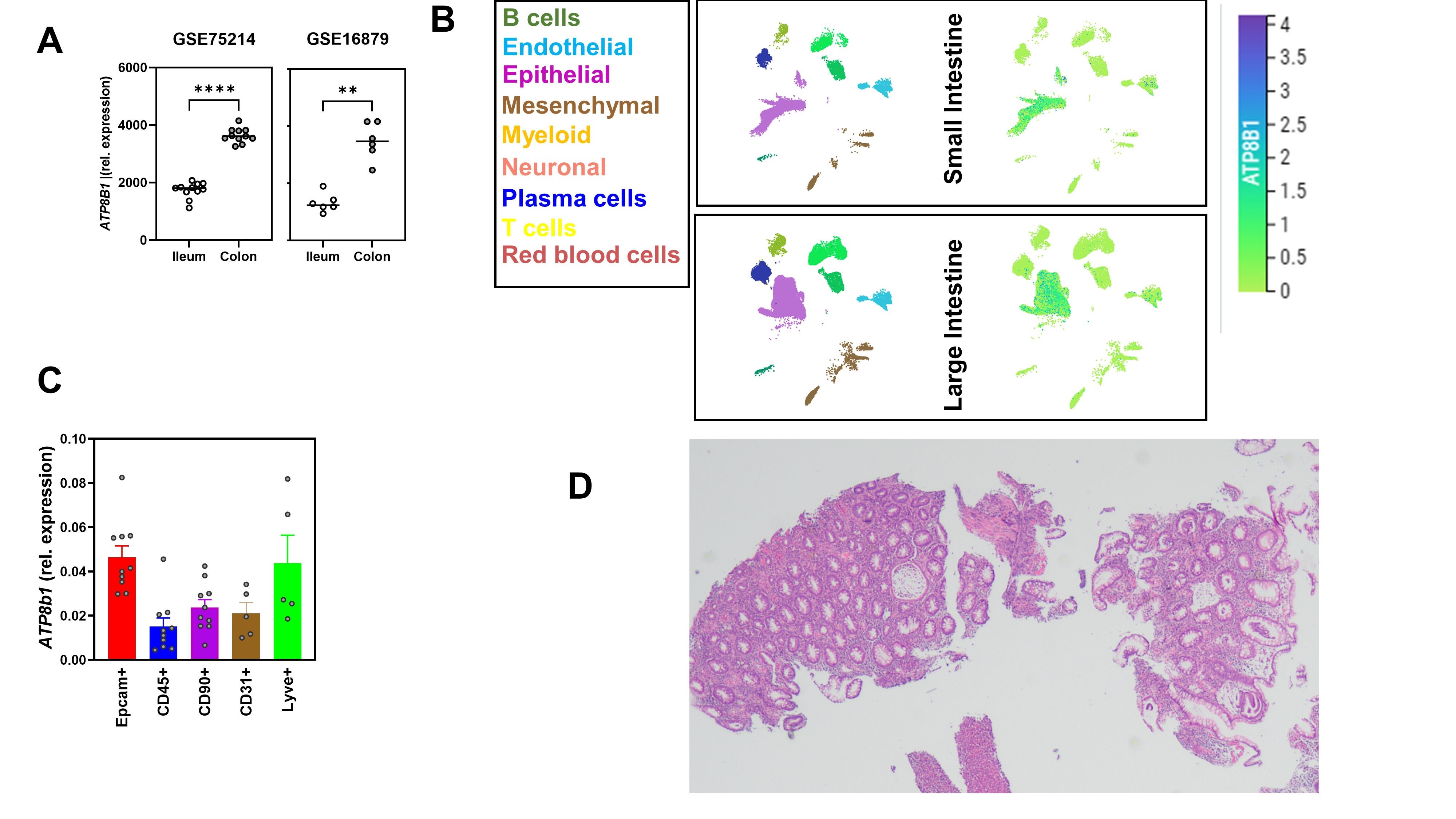

Supplement: jjae024_suppl_Supplementary_Figures_S1 [file jjae024_suppl_supplementary_figures_s1.jpeg]

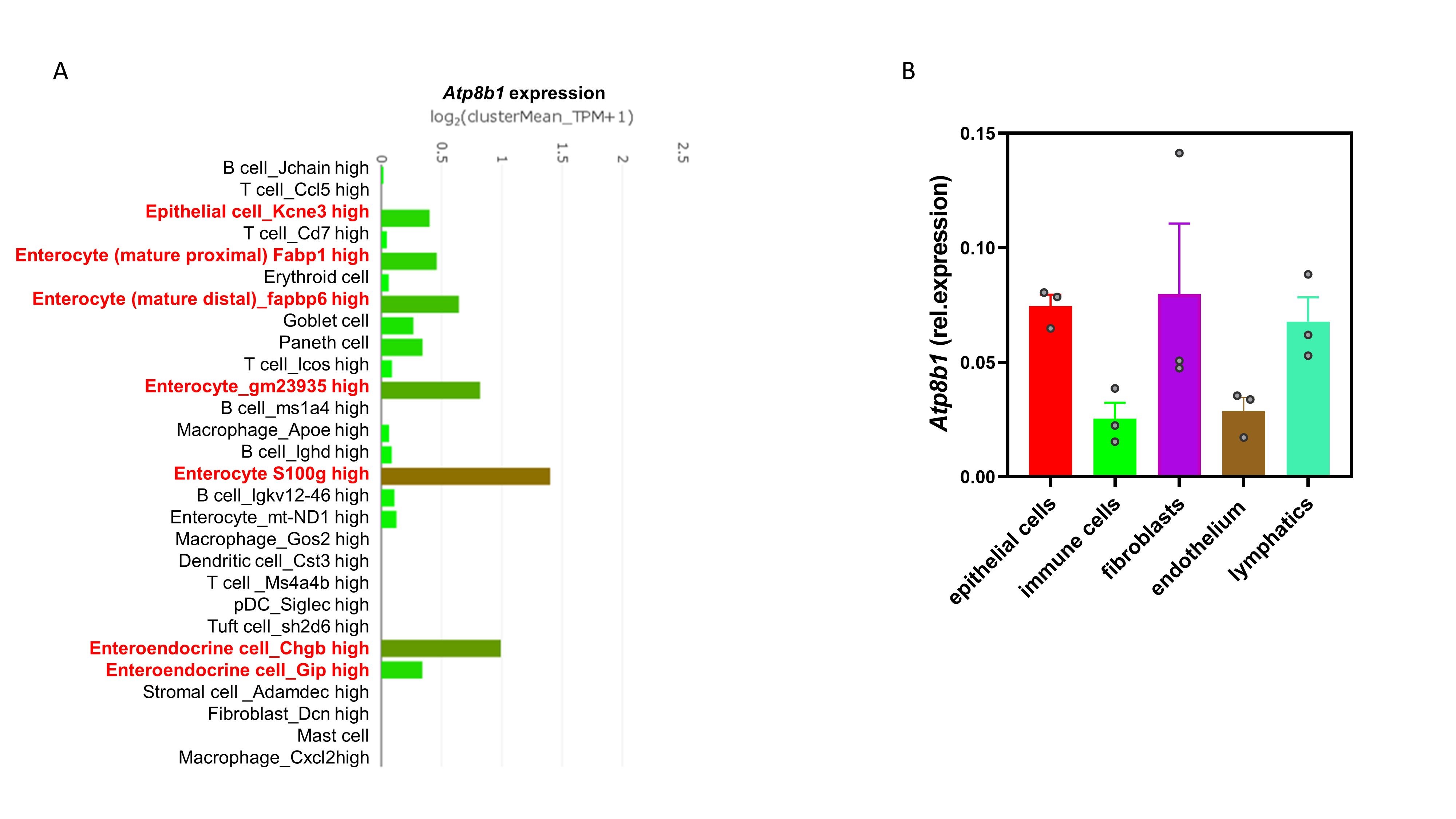

Supplement: jjae024_suppl_Supplementary_Figures_S2 [file jjae024_suppl_supplementary_figures_s2.jpeg]

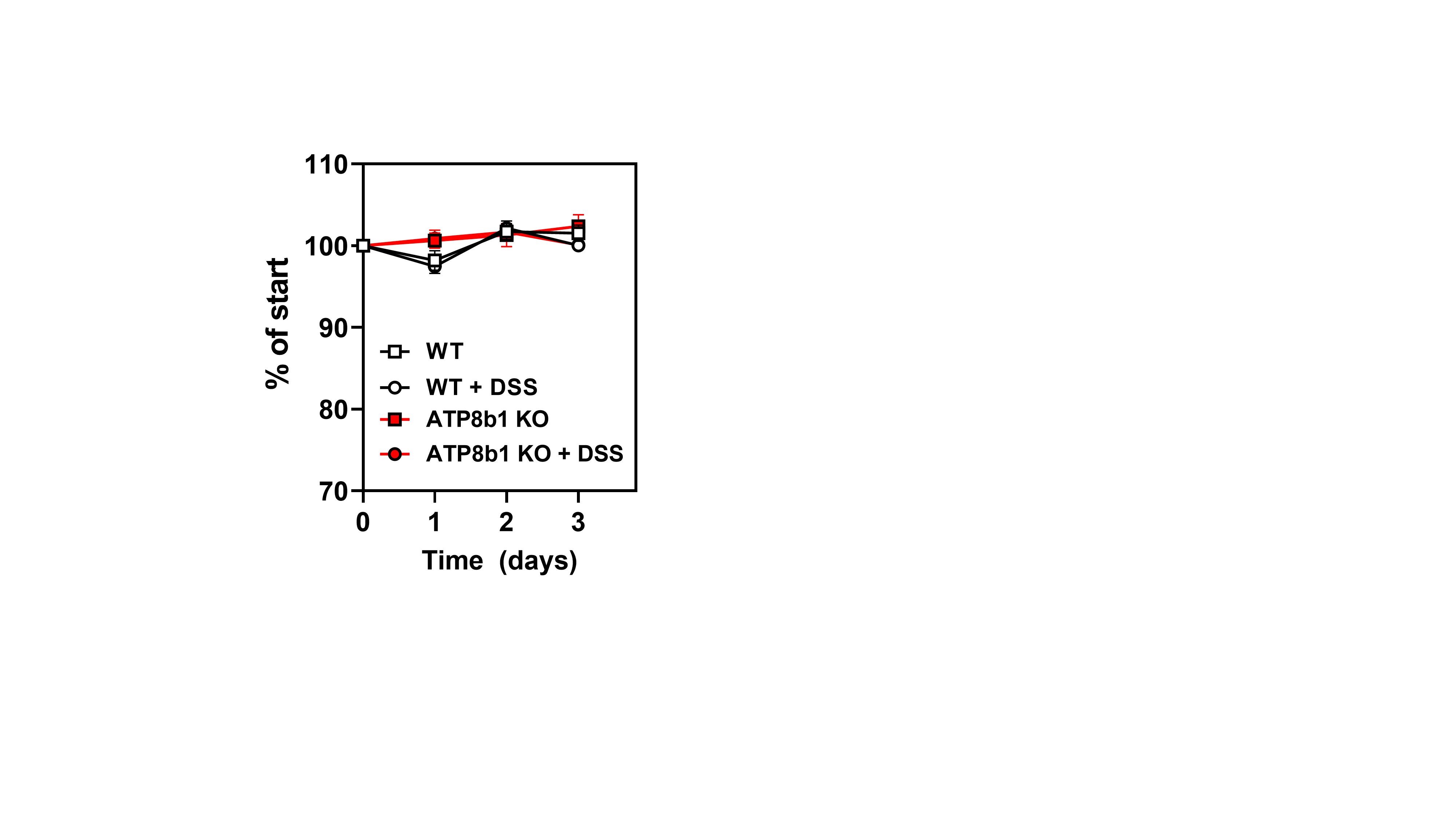

Supplement: jjae024_suppl_Supplementary_Figures_S3 [file jjae024_suppl_supplementary_figures_s3.jpeg]

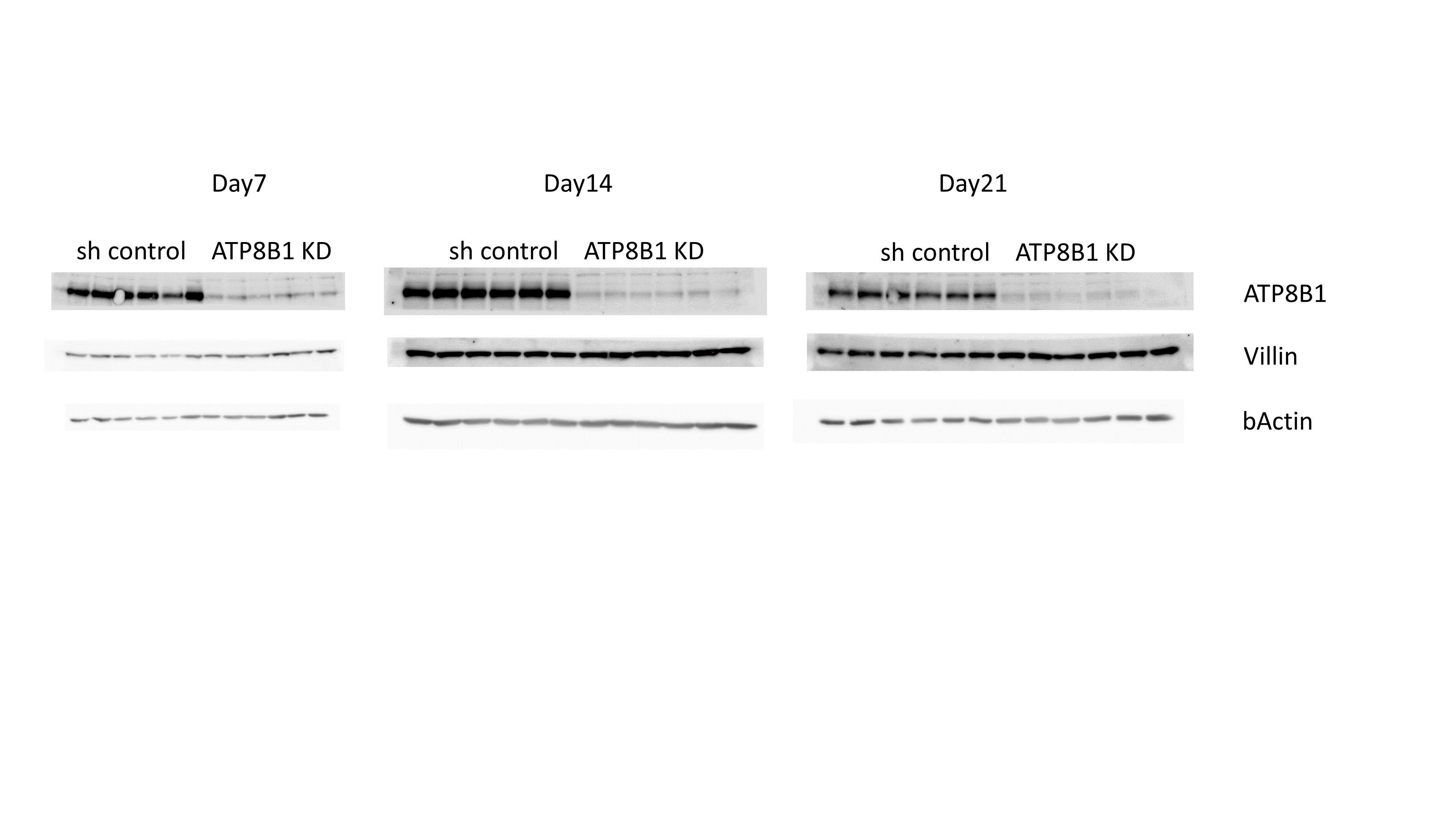

Supplement: jjae024_suppl_Supplementary_Figures_S4 [file jjae024_suppl_supplementary_figures_s4.jpeg]

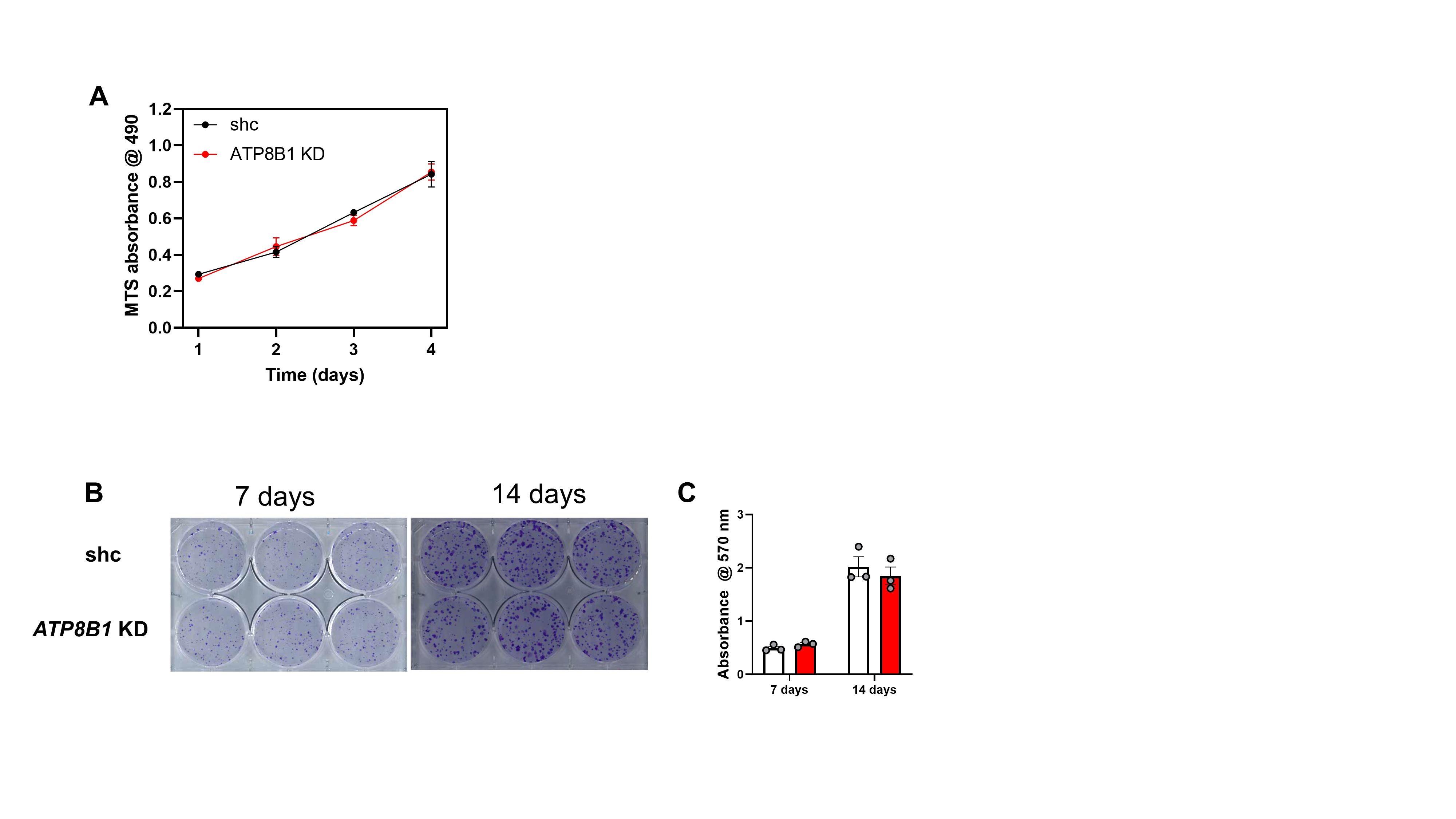

Supplement: jjae024_suppl_Supplementary_Figures_S5 [file jjae024_suppl_supplementary_figures_s5.jpeg]

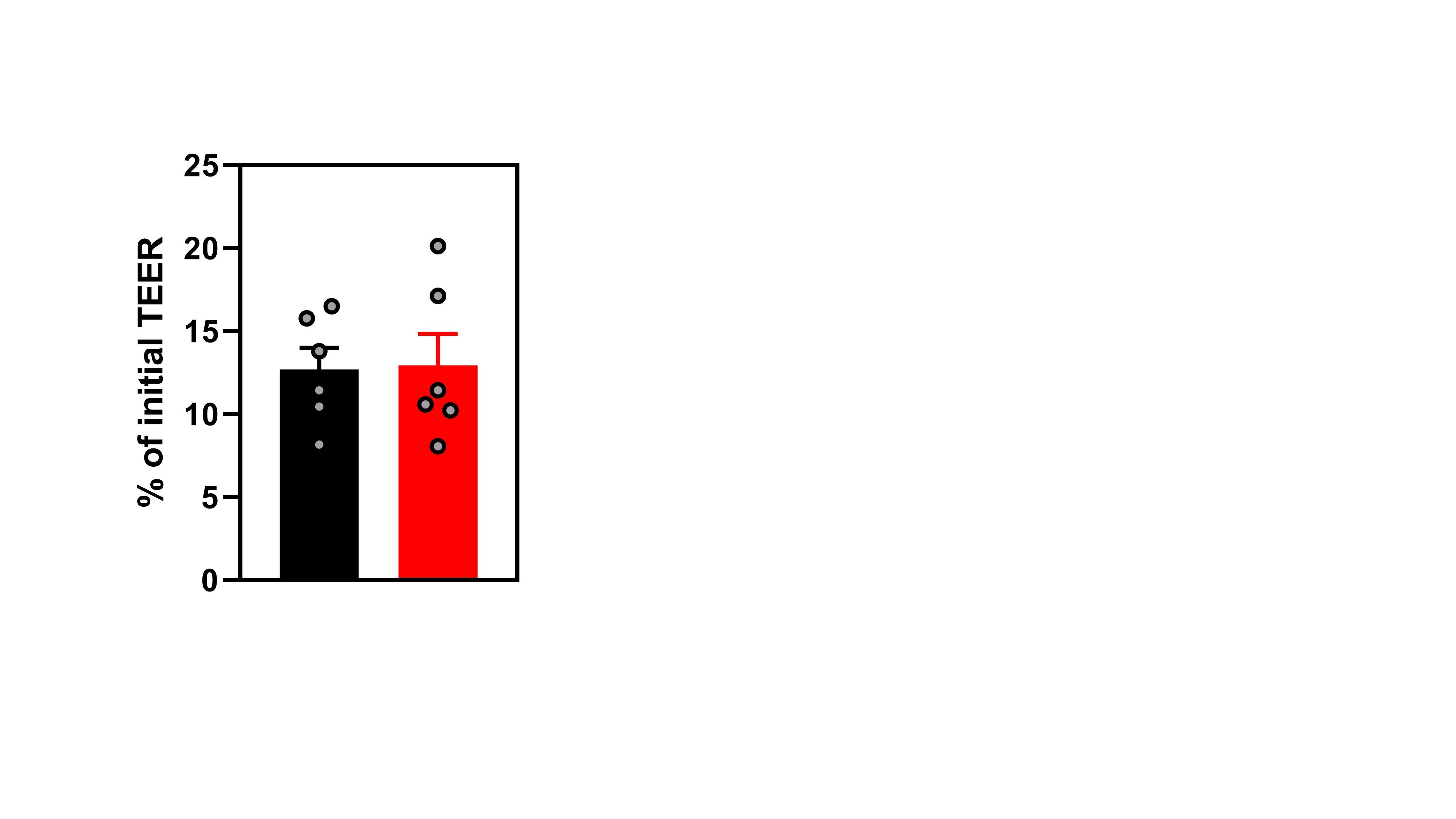

Supplement: jjae024_suppl_Supplementary_Figures_S6 [file jjae024_suppl_supplementary_figures_s6.jpeg]

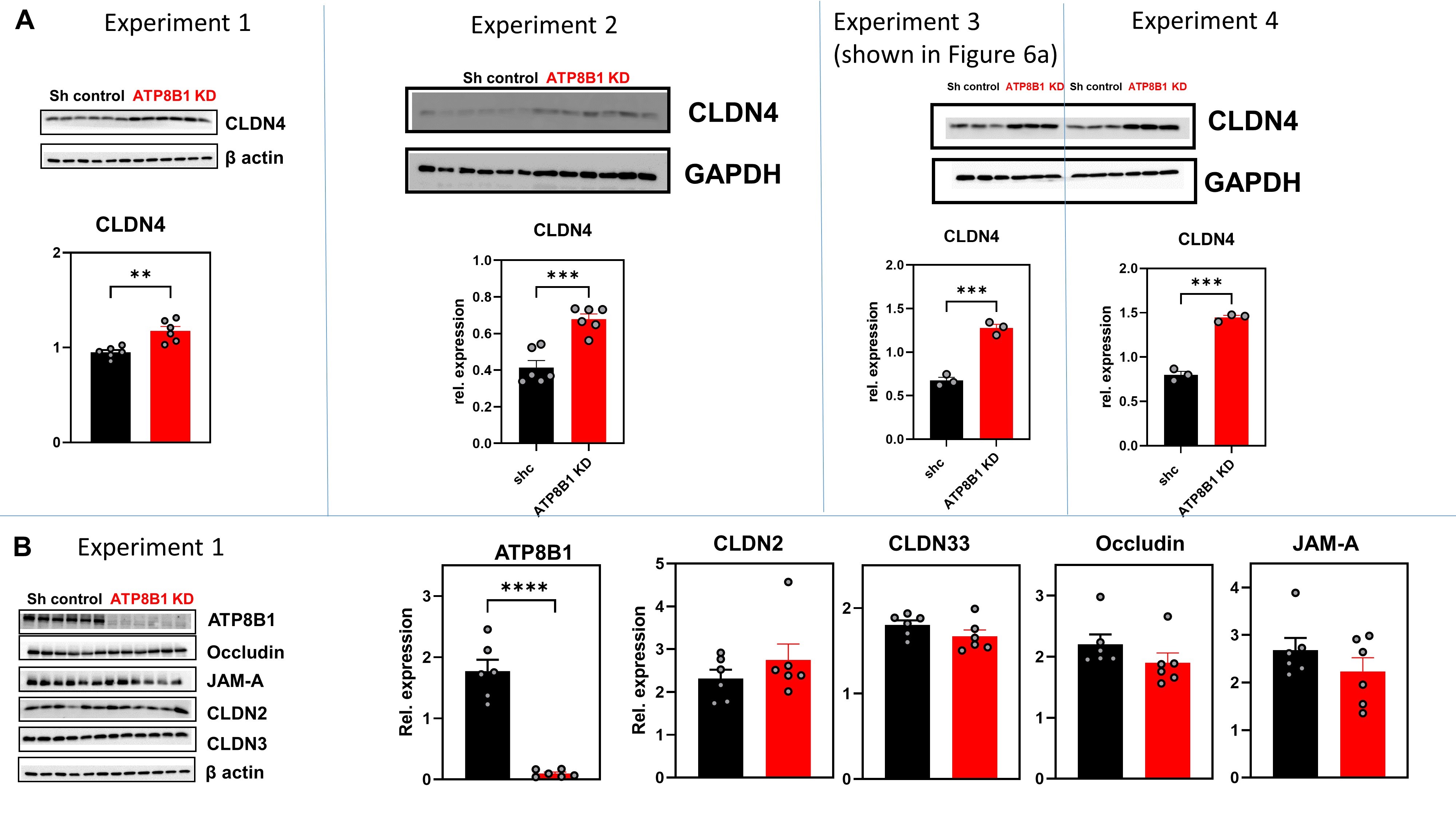

Supplement: jjae024_suppl_Supplementary_Figures_S7 [file jjae024_suppl_supplementary_figures_s7.jpeg]

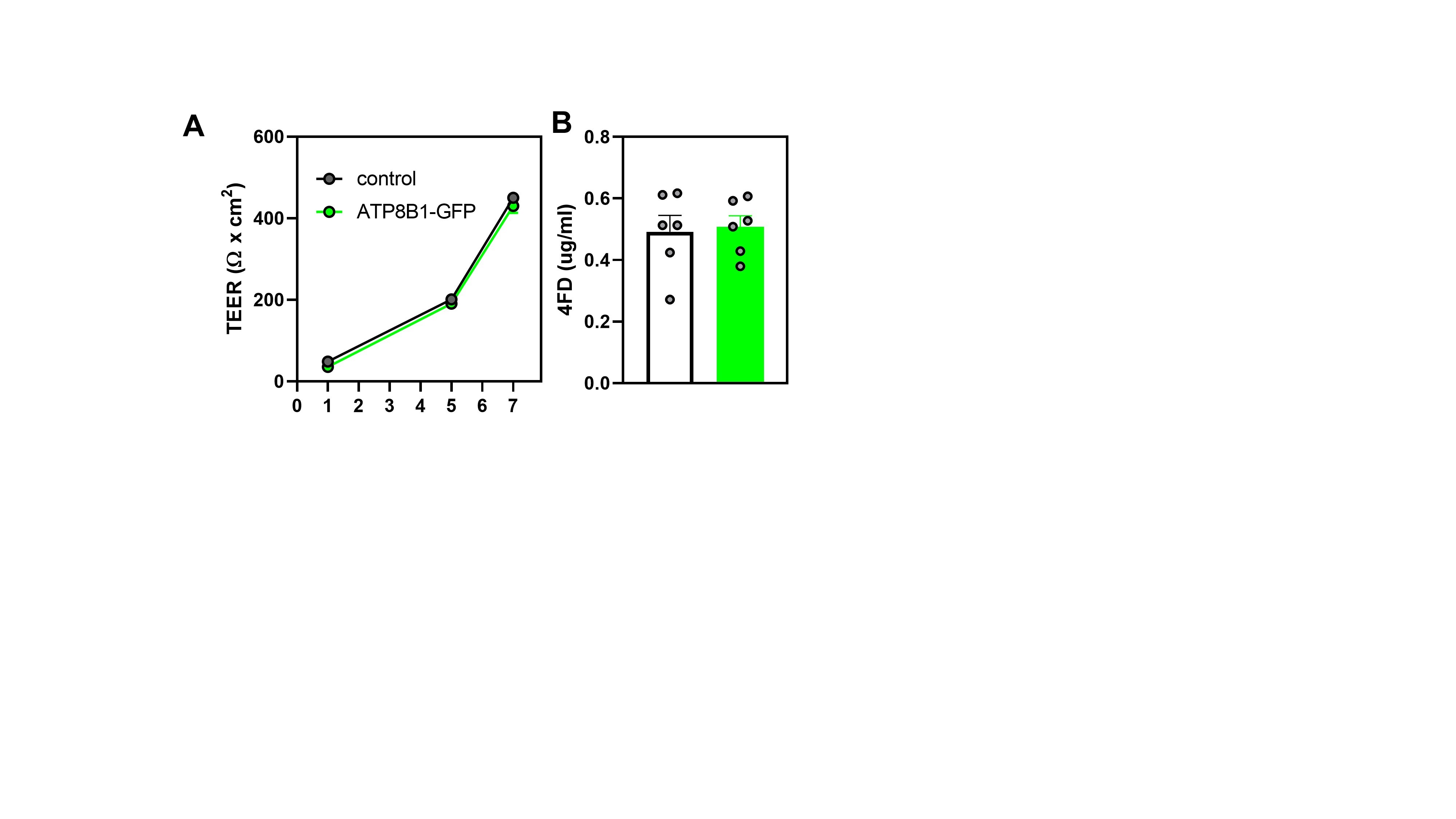

Supplement: jjae024_suppl_Supplementary_Figures_S8 [file jjae024_suppl_supplementary_figures_s8.jpeg]

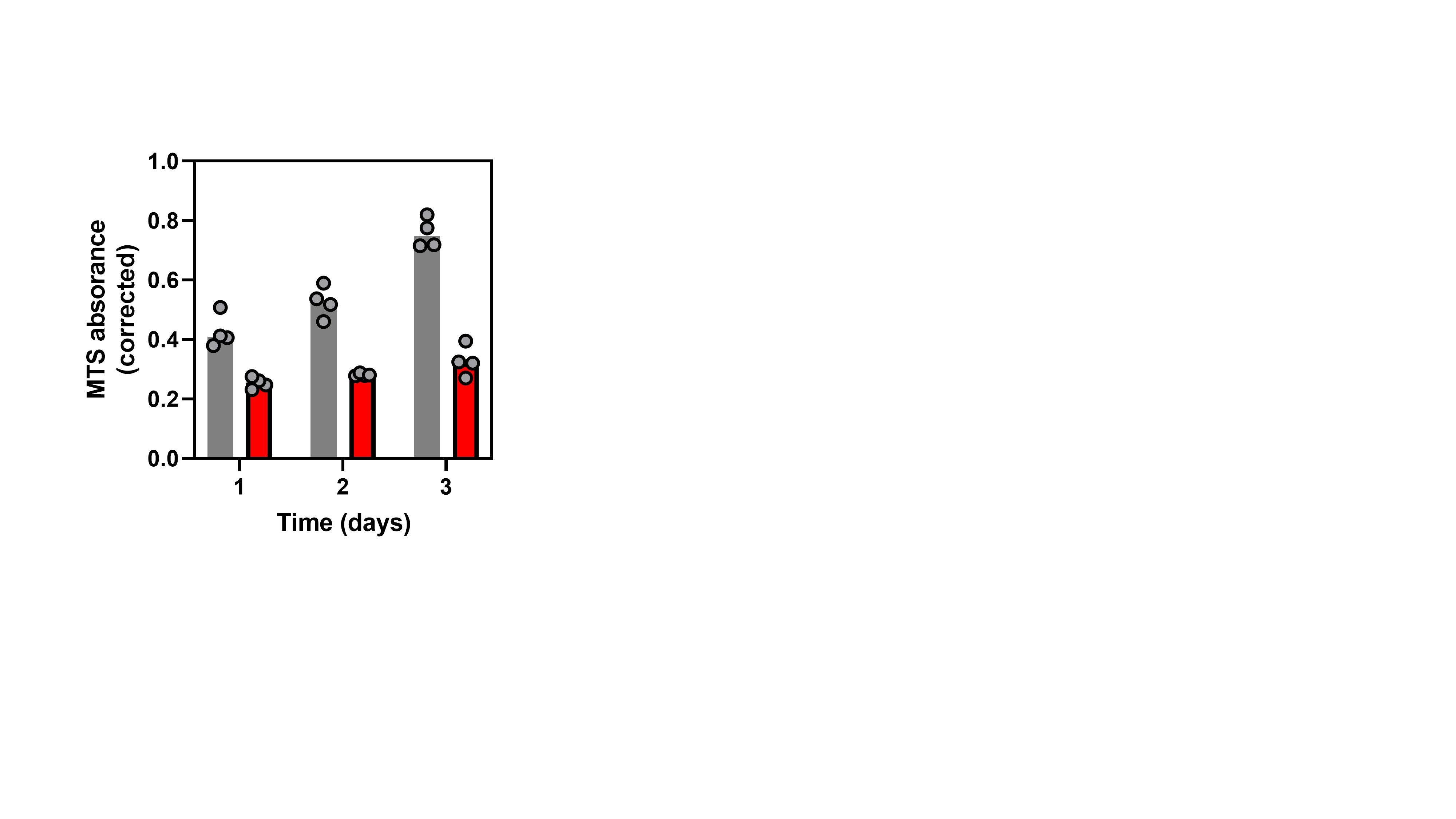

Supplement: jjae024_suppl_Supplementary_Figures_S9 [file jjae024_suppl_supplementary_figures_s9.jpeg]
